# Evaluating the iron chelator function of sirtinol in non-small cell lung cancer

**DOI:** 10.3389/fonc.2023.1185715

**Published:** 2023-06-15

**Authors:** Michael S. Petronek, Khaliunaa Bayanbold, Koffi Amegble, Ann M. Tomanek-Chalkley, Bryan G. Allen, Douglas R. Spitz, Charvann K. Bailey

**Affiliations:** ^1^ Department of Radiation Oncology, Division of Free Radical and Radiation Biology, University of Iowa, Iowa City, IA, United States; ^2^ Department of Biology, Grinnell College, Grinnell, IA, United States

**Keywords:** cancer therapy, iron metabolism, non-small cell lung cancer (NSCLC), iron, cancer biology

## Abstract

A distinctive feature of cancer is the upregulation of sirtuin proteins. Sirtuins are class III NAD+-dependent deacetylases involved in cellular processes such as proliferation and protection against oxidative stress. SIRTs 1 and 2 are also overexpressed in several types of cancers including non-small cell lung cancer (NSCLC). Sirtinol, a sirtuin (SIRT) 1 and 2 specific inhibitor, is a recent anti-cancer agent that is cytotoxic against several types of cancers including NSCLC. Thus, sirtuins 1 and 2 represent valuable targets for cancer therapy. Recent studies show that sirtinol functions as a tridentate iron chelator by binding Fe3+ with 3:1 stoichiometry. However, the biological consequences of this function remain unexplored. Consistent with preliminary literature, we show that sirtinol can deplete intracellular labile iron pools in both A549 and H1299 non-small cell lung cancer cells acutely. Interestingly, a temporal adaptive response occurs in A549 cells as sirtinol enhances transferrin receptor stability and represses ferritin heavy chain translation through impaired aconitase activity and apparent IRP1 activation. This effect was not observed in H1299 cells. Holo-transferrin supplementation significantly enhanced colony formation in A549 cells while increasing sirtinol toxicity. This effect was not observed in H1299 cells. The results highlight the fundamental genetic differences that may exist between H1299 and A549 cells and offer a novel mechanism of how sirtinol kills NSCLC cells.

## Introduction

Globally, lung cancer is one of the leading causes of cancer deaths. Approximately 80%–85% of all lung cancer diagnoses are non-small cell lung cancer (NSCLC), and the 5-year overall survival remains approximately 28% (1). Such a despondent figure indicates a need for more effective treatment. Historically, treatments for cancer consisted of radiotherapy and chemotherapy, which have had limited success, treating NSCLC (2, 3). More contemporary techniques like targeted therapy allow researchers to exploit distinctive characteristics of cancer cells for more precise treatment (4). One consistent attribute of lung cancer cells is the overexpression of sirtuin 1 and 2 (SIRT1/2) proteins compared to normal human bronchial epithelial cells where enhanced SIRT1/2 expression has been correlated with a poor prognosis in NSCLC patients (5, 6). SIRTs are class III nicotinamide adenine dinucleotide (NAD)^+^-dependent histone deacetylases that modulate senescence, cell cycle regulation, apoptosis, and oxidative stress response (7). By using NAD^+^ as a cosubstrate for their modulation of histone deacetylation, targets such as H3K9ac or non-histone targets like Foxo3a and sirtuins are critical for mediating cell survival (8). Current literature reveals that targeted inhibition of SIRT1 and 2 proteins in cancer is beneficial for constraining their growth and defense mechanisms—making them more susceptible to apoptosis.

Sirtinol is a SIRT 1 and 2 pharmacological inhibitor that was discovered during cell-based screening of *Saccharomyces cerevisiae* yeast by accessing Sir2p inhibition (9). Its potent inhibition of SIRT1 and 2 and has been investigated as an anticancer agent (10). Sirtinol induces senescence and apoptosis in MCF-7 breast cancer cells and H1299 NSCLCs (11, 12). Although sirtinol is not currently a mainline therapeutic used to treat NSCLCs, several studies show its potential for use in the clinic. For example, Fong et al. showed that sirtinol is cytotoxic to NSCLC cells in a dose-dependent manner after 24 and 48 h treatment (13). This study also showed that sirtinol reduces the proliferation of cancer cells by pausing the cell in the G1 phase of the cell cycle. Sirtinol also enhances the cytotoxicity of traditional chemotherapeutic agents like gemcitabine and cisplatin (14, 15), while reducing inflammation in normal epithelial cells, suggesting selective sensitization (16).

**Figure 1:**
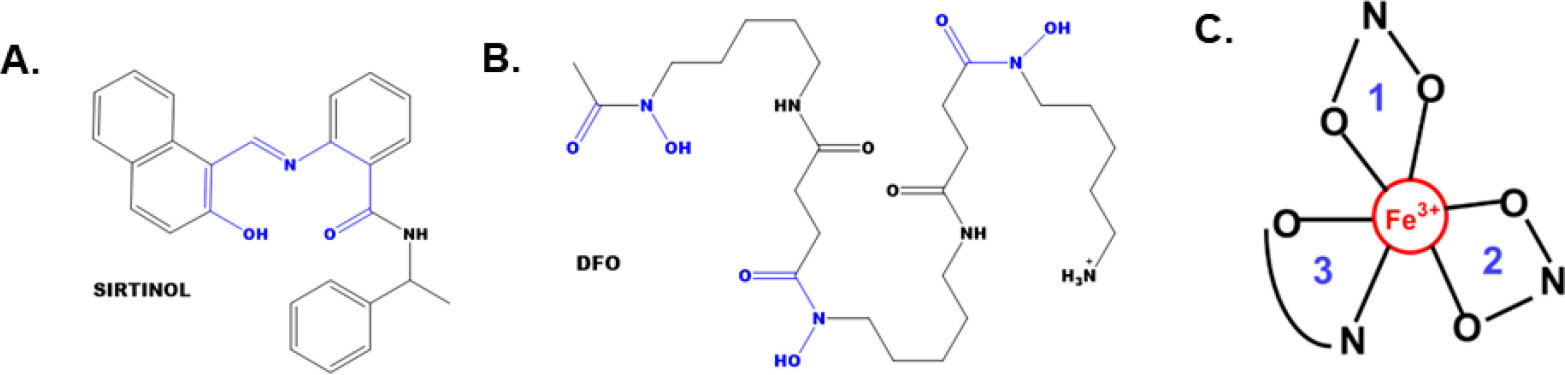
The structures of sirtinol and desferrioxamine (DFO) and its iron coordination sites. **(A, B)** The structures of sirtinol **(A)** and DFO **(B)**. The putative iron coordination site within both structures are highlighted in blue. **(C)** Tridentate, octahedral, high-spin Fe^3+^ complex formed from the binding of sirtinol to a free Fe atom. Blue numbers represent independent sirtinol molecules bound to the Fe atom.

Recently, sirtinol was also discovered to be an intracellular iron chelator (17). This observation evolved from evaluating the chemical structure of sirtinol relative to other iron-chelating molecules [*e.g.*, desferrioxamine (DFO) or deferasirox] (7). Sirtinol’s structure contains a 2-hydroxynaphthalenyl moiety that is connected to a benzamide through an aldiminic nitrogen atom (**Figures 1A, B**). This group can provide a tridentate O – N – O donor moiety that is often observed in other iron-chelating molecules. Crystallography analysis showed that sirtinol can function as a tridentate iron chelator by binding a single Fe^3+^ atom with 3:1 (sirtinol:Fe^3+^) stoichiometry to form an octahedral, high-spin (S = 5/2) Fe^3+^ complex comprised of five donor oxygen atoms and one donor nitrogen atom (**Figure 1C**) (17). This is a critical finding as iron chelation may serve as an important drug effect associated with the cytotoxicity of sirtinol. However, the biological significance of sirtinol’s iron-chelator function remains unclear. Thus, it was hypothesized that sirtinol can impair iron metabolism in NSCLC cells and this study aimed to evaluate the biological significance of the chelator function of sirtinol in NSCLC.

Iron metabolism is a critical feature of a multitude of cellular functions central to cancer progression (18). It is altered in a wide array of cancer types with cancer cells typically harboring an iron dependency as compared to their normal tissue counterparts. Iron metabolism is altered in human NSCLC tumor tissue with increased hepcidin expression (19). Increased transferrin receptor (TfR) expression has also been observed in human NSCLC tumors (20). These data suggest that NSCLC tumors accumulate iron preferentially. Iron chelation therapy may be an attractive anticancer therapeutic strategy. For example, deferoxamine has recently been shown to selectively induce mitochondrial dysfunction in cancer cells (21).

In this study, A549 and H1299 NSCLC cells were used to evaluate the chelator function of sirtinol. These cells represent two widely used NSCLC *in vitro* models with varying clinically relevant genetic backgrounds (**Table 1**). Importantly, A549 cells harbor mutations that have been observed in aggressive lung tumors. STK11 comutations as observed in the A549 cells are relatively frequent with both KRAS (54%) and KEAP1 (27%), where STK11 comutations are associated with worse clinical outcomes (27). Thus, A549 cells provide an *in vitro* model of an aggressive NSCLC tumor.

**Table 1:** Genetic differences in cell lines used in this study.

Genetic Background (mutant frequency, %)	A549	H1299
KRAS (17%) (22)	G12S mutant (23)	Wild type
STK11 (27%) (22)	Q37 mutant (24)	Wild type
KEAP1 (15%) (25)	G333C mutant (26)	Wild type

## Results

**Figure 2:**
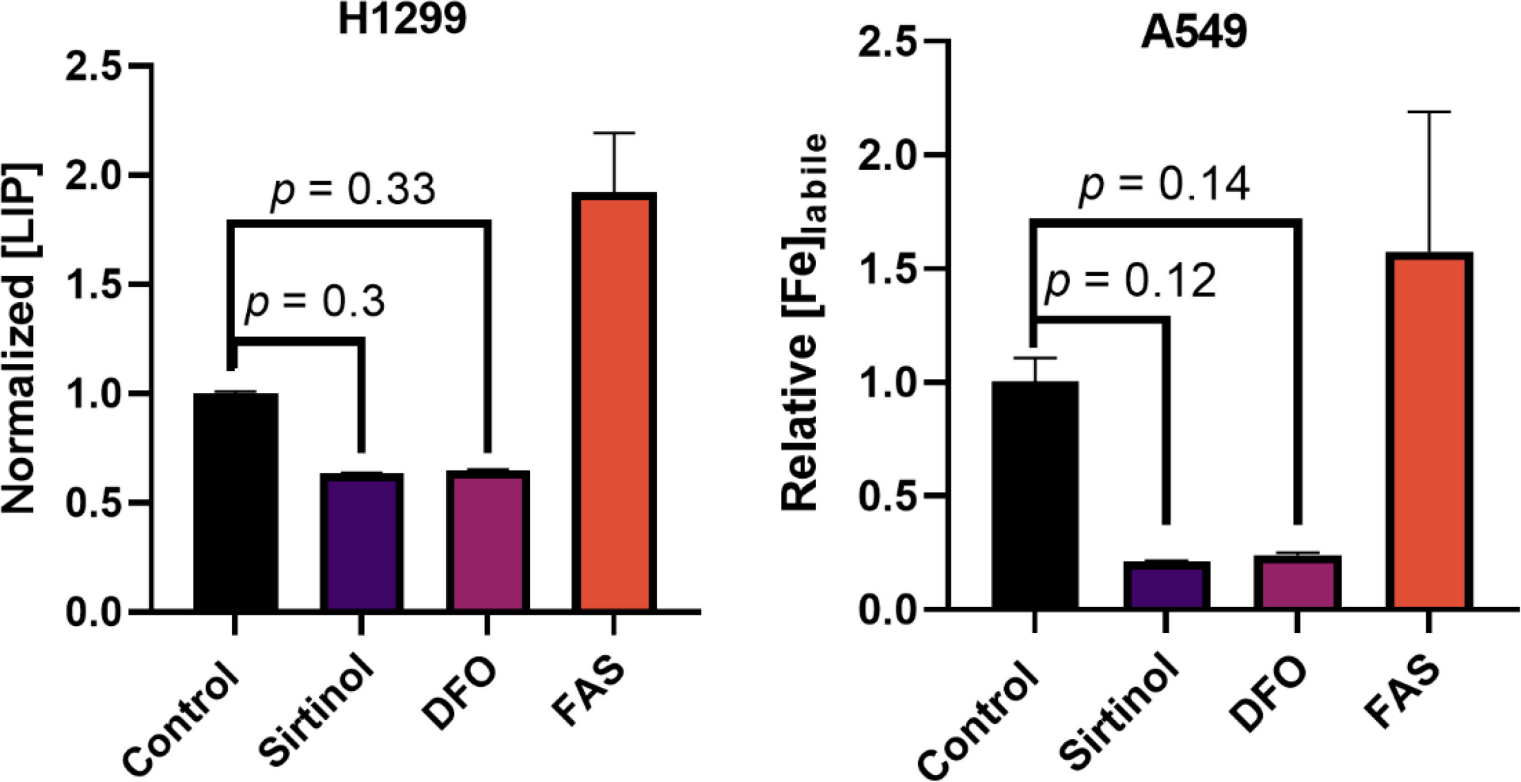
Sirtinol behaves like DFO to chelate iron in non-small cell lung cancer (NSCLC). Effects on labile iron in H1299 and A549 cells by a 50 µM 72 h treatment of sirtinol and DFO using a calcein–AM flow cytometry probe. Conversely, 3 h, 50 µM ferrous ammonium sulfate was used as a positive control. Error bars represent the mean ± SEM of *n* = 3 replicates. Statistical analysis was done using a one-way ANOVA with a *post-hoc* Tukey’s test for individual comparisons.

First, the proposed chelator function was evaluated in this *in vitro* NSCLC model. Consistent with this function, a 72 h treatment of sirtinol caused decrease in intracellular labile iron in both H1299 and A549 cells (**Figure 2**). Importantly, the decrease in labile iron by sirtinol is the same effect as observed by DFO. An interesting observation was that this effect was greater in the A549 cells (79.2% decrease) as compared to the H1299 (36.3% decrease). Thus, we have been able to recapitulate the ability of sirtinol to serve as an iron chelator to a differential effect in these genetic subtypes of NSCLC.

### Sirtinol alters iron metabolic features in non-small cell lung cancer cells

**Figure 3:**
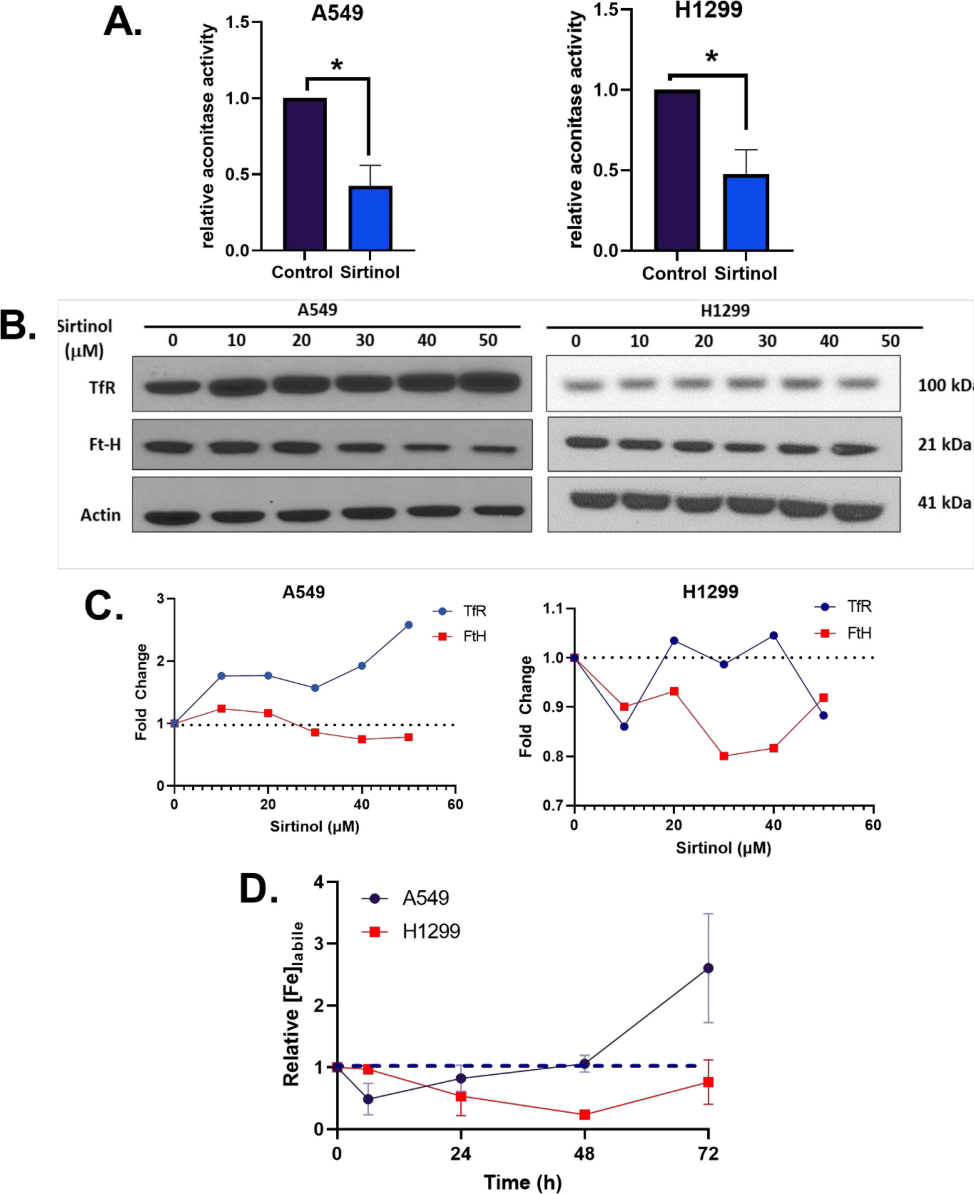
Sirtinol alters iron metabolic features. **(A)** Relative aconitase activity in A549 and H1299 cells following a 24 h treatment of 50 µM sirtinol. Aconitase activity was evaluated by measuring the rate of appearance of nicotinamide adenine dinucleotide phosphate (NADPH) at 340 nm in the presence of citrate and isocitrate dehydrogenase. Error bars represent the mean ± SEM of *n* = 3–4 experiments where *p< 0.05 using an unpaired, Welch’s T-test. **(B)** A549 and H1299 NSCLC cells were treated for 24 h with 0–50 µM sirtinol and then harvested for evaluation of the transferrin receptor (TfR) and ferritin (FtH) expression using a Western blot approach. **(C)** Western blot quantification TfR and FtH changes in A549 and H1299 cells. **(D)** A549 and H1299 NSCLC cells were treated for 6, 24, 48, or 72 h with 50 µM sirtinol and then harvested for evaluation of labile iron pool concentrations using a colorimetric ferrozine-based assay. Each timepoint was normalized to an untreated control. Dashed black line indicates baseline measures. Error bars represent mean ± SD from triplicate measures.

Because of the initial validation of the sirtinol iron chelator function, it was hypothesized that this would result in metabolic alterations associated with labile iron regulation. Aconitase was first interrogated as it is an iron-dependent tricarboxylic acid (TCA)-cycle intermediate that utilizes a complete [4Fe-4S]^2+^ cluster for the isomerization of citrate to isocitrate (28). Under conditions of limited intracellular iron availability, the cluster will be in an incomplete [3Fe-4S]^+^ form leading to enzyme inactivation (29). In both A549 and H1299 cell lines, a significant decrease in aconitase activity (>50% reduction in enzymatic activity, p< 0.05) was observed (**Figure 3A**), further indicating that sirtinol’s chelator activity can sequester iron to impair the activity of iron-dependent enzymes.

Converse to its TCA-cycle function, in an inactive [3Fe-4S]^+^ form, aconitase can function as iron-responsive protein-1 (IRP1). IRP1 responds to intracellular Fe levels to promote either stabilization or degradation of TfR and ferritin heavy-chain (FtH) mRNAs (30). When there is limited intracellular iron availability, IRP1 binds to iron response elements (IREs) to maintain homeostasis by binding at the 3^’^ end of TfR mRNA to enhance stability and promote translation (31–33) or the 5’ end of FtH to repress translation (34, 35). Interestingly, a sirtinol concentration–dependent increase in TfR protein levels and FtH repression cells was observed in A549 cells that was not apparent in H1299 cells (**Figures 3B, C**). This further supports the hypothesis that the chelator function of sirtinol disrupts iron metabolism by limiting intracellular iron availability, although the biological effects appear to present as a cell type–dependent effect. Therefore, it appears that sirtinol may be able to impair the IRP-IRE system through its chelator function; however, this requires further validation. Based on the observed iron metabolic perturbations, the temporal-dependent changes in intracellular iron were interrogated. Using this colorimetric assay, sirtinol was observed to chelate iron in both H1299 and A549 cell lines, as relative labile iron concentrations were decreased in both cell lines acutely (**Figure 3D**). In A549 cells, the labile iron pool was decreased acutely (within 6 h) while noticeable decreases did not occur in H1299 cells until 24 h sirtinol treatment was used. Interestingly, after the acute decrease in labile iron in A549, there was an apparent adaptive response where labile iron continued to increase above the basal level until 72 h. A similar trend was not observed in the H1299 cells as labile iron was decreased through 48 h until a slight increase from 48 to 72 h occurred but remained below the basal level. These results are consistent with the cell type–specific adaptive response that occurred in the A549 cells. These results further support the iron chelator function of sirtinol, and these observations may represent a fundamental difference in iron metabolic regulation and response to perturbations between NSCLC cell lines with variable genetic backgrounds.

### Sirtinol impairs iron-dependent colony formation in A549 cells

**Figure 4:**
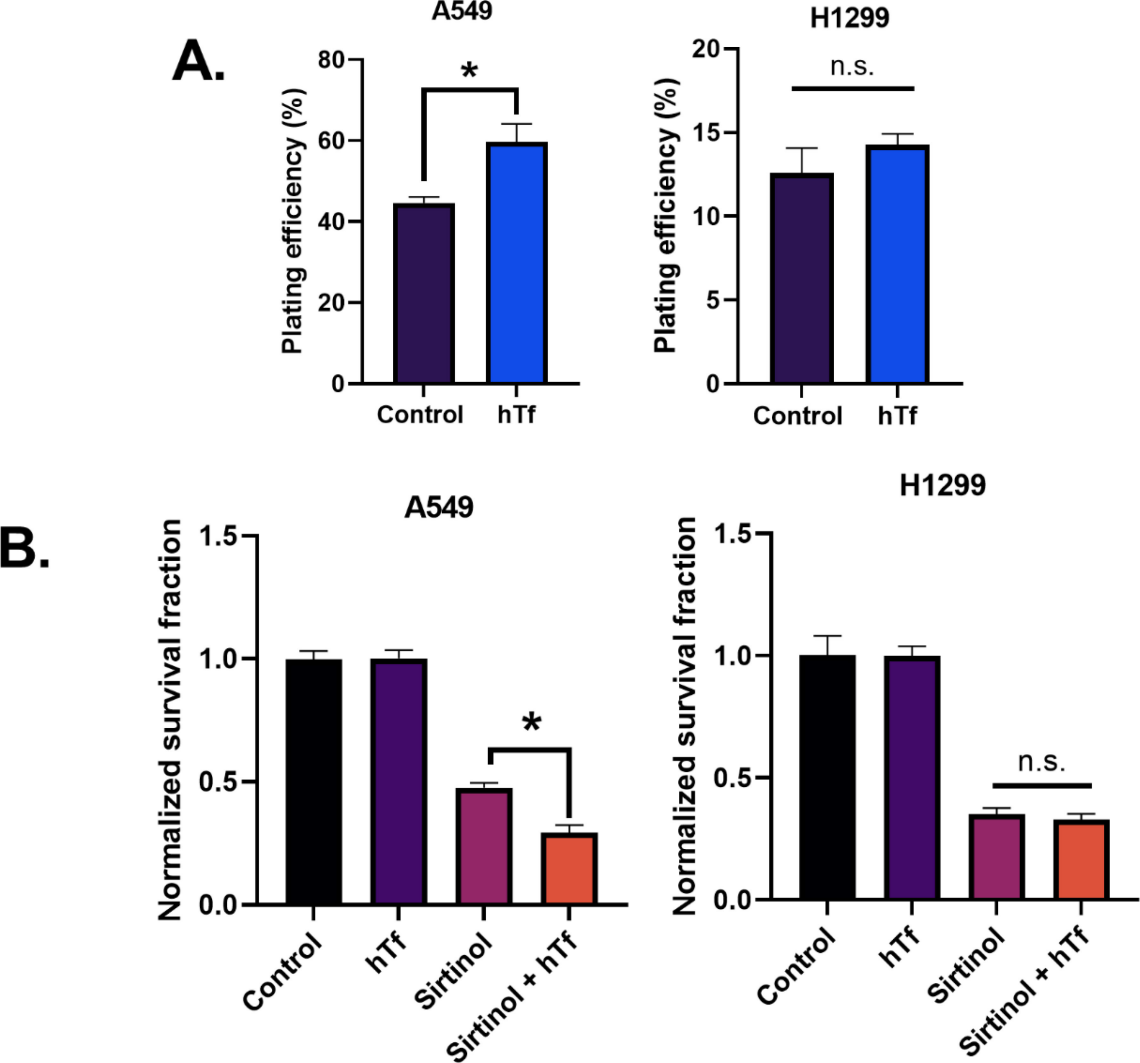
Sirtinol impairs iron-dependent colony formation in A549 cells. **(A)** Plating efficiency (%) of A549 and H1299 cells following a 24 h supplement of 200 µg ml^-1^ holo-transferrin (hTf). Error bars represent mean ± SD (*n* = 3) with *p< 0.05 using a paired, two-tailed Welch’s T-test. **(B)** Clonogenic survival of A549 and H1299 cells treated for 24 h ± sirtinol (50 µM) ± hTf (200 µg ml^-1^). Error bars represent mean ± SD (*n* = 3) with *p< 0.05 using a one-way ANOVA test.

Based upon sirtinol’s induced iron-metabolic shifts, the biological relevance of these findings was interrogated by investigating its effects on iron-dependent colony formation. First, the effects of holo-transferrin [hTf; di-ferric transferrin; Tf-(Fe^3+^)_2_] on NSCLC plating efficiency were assessed. Interestingly, a significant increase in plating efficiency (40%–60%) was observed in A549 cells following a 24 h supplementation of cell culture media with hTf while there was no effect in H1299 cells (**Figure 4A**). Thus, A549 cells exhibit a pattern of iron dependency to facilitate colony formation, while H1299 cells do not. Similarly, sirtinol enhanced cell-killing in A549 cells supplemented with hTf but had little to no effect in H1299 cells (**Figure 4B**). Therefore, the chelator function of sirtinol appears to play a role in sirtinol’s cytotoxicity but is largely context dependent.

## Discussion

This study has shown that sirtinol can function as an intracellular iron chelator to induce iron metabolic changes. Importantly, the chelator function of sirtinol has been further validated in NSCLC cells as sirtinol exhibited the same effects on labile iron as DFO. The ability of sirtinol to deplete intracellular labile iron is consistent with the previous literature that sirtinol can function as a tridentate iron chelator (17). Furthermore, sirtinol has been observed to deplete intracellular labile iron acutely leading to an adaptive response; however, the adaptive response is cell line dependent. The adaptive response that we observed, primarily in A549 cells, is consistent with decreased aconitase activity (i.e., enhanced IRP1 activation) leading to increased TfR stability and decreased FtH translation. This is likely due to the sequestering of iron leading to an incomplete [3Fe-4S]^+^ cluster in aconitase (30). Thus, it can be hypothesized that a disruption in the IRP-IRE system results in an increase in TfR stability leading to increased iron uptake *via* receptor-mediated endocytosis (36, 37). Meanwhile, the decrease in FtH expression indicates decreased iron storage capacity, as ferritin is the primary iron storage enzyme of the cell (38). Further experiments are required to elucidate the effects of sirtinol on the IRP-IRE system. However, an adaptive response to increase iron uptake and decrease storage would explain the temporally dependent increases in labile iron observed for extended sirtinol treatments in A549 cells. The increase in labile iron at 72 h observed using ferrozine is slightly contradictory to the calcein-AM results. This is likely due to the nature of each individual assay as calcein-AM can bind iron irrespective of its oxidation state but is unable to remove iron from other complexes, while ferrozine is Fe^2+^-dependent and utilizes ascorbic acid to convert all of the iron in a highly acidic (pH ≈ 4–4.5) solution to convert all of the iron to Fe^2+^. Thus, the ferrozine results at 72 h may be more reflective of the total iron accumulated within the cells in addition to the iron bound to sirtinol, while the calcein-AM results are more likely to reflect the amount of iron remaining to be chelated after treatment. This hypothesis is based on the notion that sirtinol iron binding permits iron recycling allowing for the iron to be reduced to Fe^2+^ under the acidic conditions. This would be consistent with previously reported literature that a sirtinol-Fe^3+^ complex can enhance oxidation of the DCFH fluorescent probe, indicating that this is a redox-active chelator capable of catalyze redox reactions (39). Based on these observations, the iron-chelator capacity of sirtinol previously described chemically appears to have biological importance and is a drug effect that should be considered when evaluating the therapeutic effects of sirtinol (17).

An unexpected discovery is the iron-dependent differences observed between A549 and H1299 NSCLC cells. Consistently, a more pronounced iron-metabolic disruption has been observed in the A549 cells throughout this study. A major, correlative difference between these two cell lines is their KRAS/STK11/KEAP-1 mutational status. A549 cells are KRAS/STK11/KEAP-1 mutant cells resulting in constitutive activation of NRF2 (40, 41) while H1299 cells are not (42). This is of particular interest because KEAP-1 mutant NSCLC cells, such as A549, are particularly aggressive and exhibit therapy resistance as patients with tumors harboring this mutational profile appear to have worse clinical outcomes (42–44). A recent retrospective study of NSCLC patients showed that KEAP-1 was a negative prognostic marker in advanced-stage (stage IIIB–IV) tumors (HR = 1.40, 95% CI: 1.23–1.61, *p<* 0.001, N = 4,779) (44). Therefore, strategies aimed at enhancing clinical responses in mutant tumors may be of critical importance in managing NSCLC. Interestingly, we have observed that STK11/KRAS/KEAP-1 mutant A549 cells exhibit a pattern of iron-dependent clonogenicity that the chelator function of sirtinol can exploit while the H1299 tumors do not. While this finding is intriguing, it also represents a significant limitation of our study. Because of the concurrent mutations observed in the A549, it is currently unclear to what extent each mutation contributes to the differential iron metabolic regulation observed in these cells. Future studies should be designed to evaluate each of these mutations independently and assess their impacts on iron metabolic regulation.

## Conclusions

In summary, it has been observed that the chemical iron-chelator function of sirtinol has biological consequences. It has been shown that sirtinol can chelate iron in NSCLC cells leading to a decrease in labile iron acutely and a cell line–specific adaptive response characterized by a decrease in aconitase activity leading to a shift toward IRP1 activation, enhanced TfR stability, and repressed FtH translation. Intriguingly, KRAS/STK11/KEAP-1 mutant cells exhibit iron-dependent colony formation that can be inhibited by sirtinol, which does not occur in H1299 cells. Overall, it can be concluded that the chelator function of sirtinol has context-dependent biological consequences that may contribute to its toxicity in NSCLC.

## Materials and methods

### Cell culture

A549 and H1299 cells were grown to 80% confluence before experimentation at 21% O_2._ Cells were treated with sirtinol prepared in 50mM stocks in DMSO and stored at −80°C for the appropriate concentration and time. For iron supplementation, 10 mg ml^-1^ of human holo-transferrin (T0665 Sigma-Aldrich, St. Louis, MO) prepared in H_2_O was added directly to the cell culture media at a concentration of 200 µg ml^-1^. For experiments testing the combination, both sirtinol and holo-transferrin were added simultaneously. For colony formation assays, cells were treated, washed, and trypsinized. Following trypsinization, cells were counted and plated as single cells in a 6-well dish (≈500 cells per well). Cells were left undisturbed for 7–10 days to allow for colony formation. Colonies were then washed with 70% EtOH for fixation and stained with Coomassie blue. Stained colonies (≥50 cells) were counted under a microscope.

### Labile iron pool measures with flow cytometry

Intracellular labile iron pool measures were performed using a Calcein-AM fluorescent dye. Cells were harvested by trypsinization. After cell harvesting, cell pellets were washed in phosphate buffered saline (PBS) and then resuspended in 500 nM Calcein-AM diluted in PBS. Samples were incubated for 15 min at 4% O_2_ (37°C, 5% CO_2_). Following incubation, extracellular Calcein-AM was removed by washing with PBS, and cells were resuspended in 1 ml PBS. Following incubation 10,000 cells were analyzed on an LSR II Flow Cytometer (BD Biosciences; λ_ex_ = 488 nm, λ_em_ = 515/20 nm). The labile iron pool was quantified using the following formula:


$$\text{relative LIP }\left(\text{A}.\text{U}.\right)=\;{\left(\frac{MFI_{treatment}}{MFI_{control}}\right)}^{-1}$$

An inverse normalization was done to approximate the labile iron pool because calcein-AM functions as a “turn-off” probe.

### Colorimetric labile iron assay

Labile iron and total iron concentrations were done using a ferrozine-based colorimetric assay. Cells were homogenized in 1X RIPA lysis buffer (Sigma-Aldrich; R0278). Cells were centrifuged at maximum speed for 10 min to remove cell debris, and 100 µl of the supernatant was then diluted 1:1 in ferrozine buffer (5 mM ferrozine, 1.25 M ammonium acetate, and 10 mM ascorbate) and centrifuged again at maximum speed for 10 min to remove any protein aggregates. This step is critical as the acidic nature of the buffer (pH ≈ 4–4.5) will result in protein aggregation that can cause the samples to become cloudy and alter the absorbance profile, resulting in an experimental artifact. Thus, samples with remaining protein aggregates were removed from analysis. The supernatant was then placed in a single well of a clear 96-well plate. Following dilution, the 96-well plate was evaluated for the formation of a Fe2+-ferrozine complex by monitoring the absorbance at 562 nm and Fe concentration was calculated using Beer’s Law:


$${\text{A}}_{562}\left(\text{A}.\text{U}.\right)={\text{ϵ}}_{562}*\left[\text{Fe}\right]*\text{L}$$

where A_562_ is the measured absorbance at 562 nm, ϵ562 is the molar extinction coefficient for a Fe^2+^-ferrozine complex = 27,900 M^-1^ cm^-1^, [Fe] is the calculated Fe concentration (M), and L is the pathlength for 200 µl of liquid ≈ 0.55 cm.

### Western blotting

Total protein (25 μg) was electrophoresed on a 4%–20% gradient gel (Bio-Rad) at 150 V for approximately 1.5 h. The separated proteins were transferred onto polyvinylidene difluoride (PVDF) membranes (Millipore, Billerica, CA) and non-specific binding was blocked using 5% non-fat dry milk in PBS-Tween (0.2%) for 1 h at room temperature. The membranes were incubated with primary antibodies (ferritin-heavy chain, 1:1,000 from Abcam, Cambridge, MA, TfR, 1:1,000, Invitrogen, Camarillo, CA) at 4°C overnight. B-actin served as a loading control (1:4,000; Sigma-Aldrich). Following three 5 min TBS-Tween washes, the membranes were probed with secondary antibodies (mouse anti-rabbit; 1:10,000; Sigma-Aldrich, St. Louis, MO) that were conjugated with horseradish peroxidase for 45 min. The washed membranes were incubated with a Super Signal West Pico Chemiluminescent Substrate (Thermo Scientific, Rockford, IL) and exposed to CareStream BioMax MR Film (CareStream Health, Rochester, NY). Quantification of Western blots were performed in ImageJ.

### Aconitase activity

Exponentially growing cells were scraped and frozen as dry pellets until assayed for total aconitase activity adapted from as previously described (45). Briefly, cell pellets were resuspended in 50 mM Tris-HCl, pH 7.4 with 0.6 mM MnCl_2_, and 5 mM Na-citrate and sonicated 3 × 10 s each. Protein was quantified by the Lowry method (46). Aconitase activity was measured as the rate of appearance of NADPH (at 340 nm; Beckman DU 800 spectrophotometer, Brea, CA) for 45 min during the reaction of 200 μg total sample protein with 200 μM NADP+ and 10 U isocitrate dehydrogenase.

## Data availability statement

The original contributions presented in the study are included in the article/supplementary material. Further inquiries can be directed to the corresponding authors.

## Author contributions

MP and CB performed study designs; MP, CB, KB, KA, and AT-C performed experiments; MP, CB, KB, KA, AT-C, BA, and DS performed data analysis; MP and CB prepared the manuscript; MP, CB, KB, KA, AT-C, BA, and DS contributed to editing. All authors contributed to the article and approved the submitted version.
